# The association between closed-eye unipedal standing and the risk of cognitive impairment in the elderly: a 7-year community-based cohort study in Wuhan, China

**DOI:** 10.3389/fnagi.2024.1308151

**Published:** 2024-01-26

**Authors:** Shiwei Wang, Peng Guo, Chengjing Huang, Yuqian Zhang, Bing Xiang, Jing Zeng, Feng Zhou, Xinyan Xie, Yan Guo, Mei Yang

**Affiliations:** ^1^School of Public Health, Wuhan University of Science and Technology, Wuhan, Hubei, China; ^2^Integrated Health Section, Wuhan Jiang'an Center for Disease Control and Prevention, Wuhan, Hubei, China; ^3^School Health Center, Wuhan Centers for Disease Control and Prevention, Wuhan, Hubei, China

**Keywords:** cognitive impairment, closed-eye unipedal standing, cohort study, restricted cubic spline, the elderly

## Abstract

**Objectives:**

The prevention of cognitive impairment in the elderly is one of the public health priority areas. However, the relationship between closed-eye unipedal standing and cognitive impairment remains unclear.

**Methods:**

This study was conducted on a group of elderly individuals from a community, using a prospective cohort study design. Participants were monitored for 7 years and were diagnosed with new-onset cognitive impairment. Logistic regression models and restricted cubic spline (RCS) were used to investigate the relationship between closed-eye unipedal standing and cognitive impairment. Stratified analysis by baseline characteristics were also performed.

**Results:**

At baseline, 1,652 people aged 65 years or older were enrolled. Ultimately, 880 participants completed the follow-up and 155 (17.61%) of them satisfied the diagnostic criteria for cognitive impairment at follow-up. Compared to the closed-eye unipedal standing low group as the reference, the middle (OR = 0.601, 95% CI: 0.396–0.911) and high (OR = 0.508, 95% CI: 0.329–0.785) groups had significantly lower cognitive impairment risks. RCS analysis indicated a linear relationship (*P*_non − linear_ = 0.177), with a reduced risk of developing cognitive impairment when the duration of closed-eye unipedal standing was exceeded ~2.920 s. Stratified analysis showed that for female, aged 70 years or younger, with 3 or more years of education, without lack of exercise and without falls within 1 year subgroup, the elderly in the high group of closed-eye unipedal standing had significantly reduced cognitive impairment risks.

**Conclusion:**

Among the elderly population, closed-eye unipedal standing duration was linearly and negatively associated with the cognitive impairment risk. The closed-eye unipedal standing duration might be a predictive index for cognitive impairment in the elderly.

## 1 Introduction

Cognitive impairment is characterized by a decline in one or more cognitive abilities due to various causes, such as reduced language skills, memory loss or even severe dementia, and other psychiatric disorders (Koder, 1998; American Psychiatric Association, 2013; Hu et al., 2021). In China, more than 360,000 people are diagnosed with cognitive impairment each year (Pu et al., 2023), and it is estimated that the total number of people with cognitive impairment in China will reach up to 48.68 million by 2060 (Prince et al., 2016). With the extension of the population's life expectancy, the number of individuals experiencing cognitive impairment has dramatically increased. The prevalence of mild cognitive impairment among Chinese individuals aged 60 years or older is ~14.71%. Furthermore, the annual transition rate from cognitive impairment to dementia is reported to be in the range of 10–30%, which is much higher than that in the elderly individuals with normal cognitive function (1–3%) (Jia et al., 2020). The prevention of cognitive impairment in the elderly is one of the public health priority areas.

The existing evidence for the elderly indicates that physical inactivity is a specific potential variable risk factor that can promote the development of cognitive impairment (Sabia et al., 2017; Livingston et al., 2020). Approximately 3% of cognitive impairment can be prevented by increased physical activity (Li, 2013; Lourenco et al., 2018; Livingston et al., 2020). Given the current dearth of effective treatments for cognitive impairment, identifying potential risk factors for cognitive impairment and dementia, especially those that can be modified such as physical activity, is an effective way to prevent cognitive decline.

In the absence of any visual reference, closed-eye unipedal standing relies solely on the balance receptors in the vestibular organ of the brain and the coordinated movement of the muscles throughout the body to maintain the center of gravity with a single foot supported (Springer et al., 2007). The duration of closed-eye unipedal standing is an important indicator of physical fitness for the elderly (Büchel et al., 2021). During closed-eye unipedal standing, the combined action of the vestibular system and proprioceptors plays a crucial role. The vestibular system is responsible for sensing acceleration and angular changes in the head to maintain balance (Henry and Baudry, 2019). Additionally, the proprioceptors provide important inputs for balance control by sensing information about the body's joint angles, muscle tone, and skin contact (Martínez-Amat et al., 2013). The prefrontal cortex of the brain is involved in regulating attention, somatic movements, and executive functions. Recent studies have shown that single-leg standing can activate the prefrontal cortex to improve cognitive performance (Sugihara et al., 2021). In addition, Morenilla et al. (2020) concluded that balance or cognitive performance decreased in older adults when a cognitive task was performed simultaneously with a postural control task (i.e., dual-task paradigm). Vellas et al. (1997) found that participants with abnormal balance in single-leg standing also scored worse on subjective health status, humor, and neurocognitive function. These evidences imply that common areas of the brain may be used for postural control and cognition. Without the aid of visual sense, closed-eye unipedal standing can accurately measure the relationship between postural control and cognitive function. It is rational to hypothesize that the duration of closed-eye unipedal standing might be a predictive index of cognitive impairment.

Therefore, we conducted a community-based cohort study among the elderly to investigate the relationship between closed-eye unipedal standing and the risk of cognitive impairment by tracking participants over a 7-year period. Optimizing the duration of closed-eye unipedal standing to prevent cognitive impairment may help in obtaining the maximum benefit.

## 2 Methods

### 2.1 Study population

**Figure 1:**
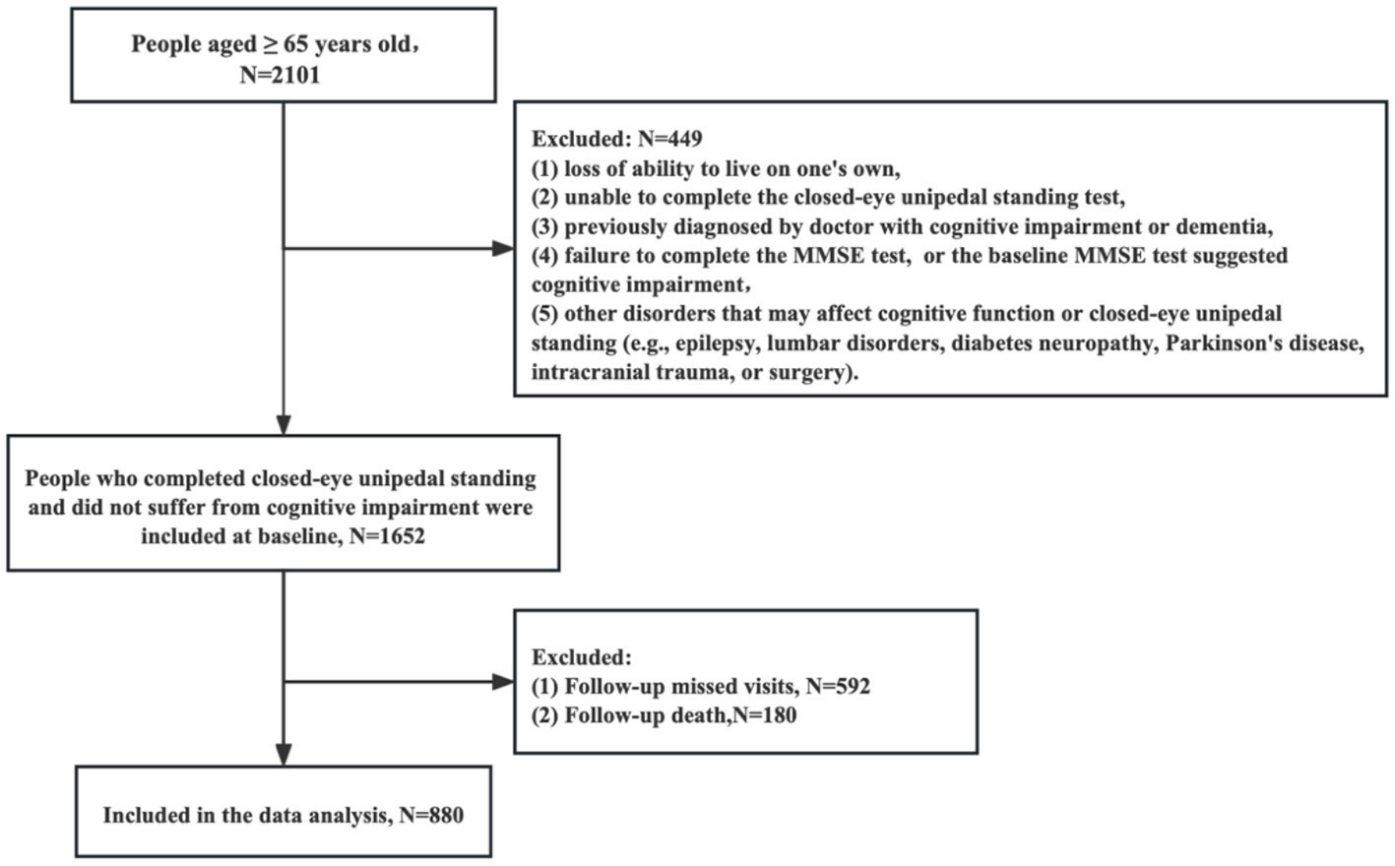
A flow chart of participant selection for this study.

Our study was a community-based prospective cohort study fousing on elderly individuals aged ≥ 65 years. In October 2015, a total of 2,101 participants were interviewed in Wuhan, Hubei Province, Central China, using a multistage stratified random sampling method. First, seven districts were randomly selected from the 17 districts in Wuhan City. Then, in each district, three to five communities were randomly recruited. In each community, 60–100 participants were randomly recruited through the Elderly Health Management Information System in Wuhan City, Hubei Province. The study staff, consisting of community doctors and nurses, were trained to use a uniform questionnaire and standardized survey terminology to collect baseline and follow-up data. At baseline, all participants self-reported their demographic characteristics and medical history (gender, age, height, weight, smoking status, years of education, and cardiovascular and cerebrovascular disease), and were required to complete the closed-eye unipedal standing test and the Mini-Mental State Examination (MMSE) test. The exclusion criteria included participants who (1) lost the ability to live on one's own, (2) were unable to complete the closed-eye unipedal standing test, (3) had been previously diagnosed by doctor with cognitive impairment or dementia, (4) failed to complete the MMSE test, or the baseline MMSE test suggested cognitive impairment, and (5) had other disorders that may affect cognitive function or closed-eye unipedal standing (e.g., epilepsy, lumbar disorders, diabetes neuropathy, Parkinson's disease, intracranial trauma, or surgery). Finally, 1,652 participants were included in our baseline study (Figure 1).

Studies involving participants were reviewed and approved by the institutional review board of Wuhan University of Science and Technology (Reference Number: 2023120). All participants provided written informed consent for their participation. The study was conducted in accordance with the Declaration of Helsinki guidelines.

### 2.2 Follow-up

The follow-up survey was conducted 7 years after the baseline in March 2023 in the same manner as the baseline examination. With 592 lost to follow-up and 180 deaths, 880 participants were finally included in this study. The mortality data were obtained from the Wuhan Death Surveillance System. Missing visitors were defined as those whose whereabouts were not known after at least three home searches, telephone contacts, or contact with community workers.

### 2.3 Cognitive assessment

The MMSE test was used to screen cognitive impairment at baseline and follow-up (Pangman et al., 2000; Arevalo-Rodriguez et al., 2015). The Chinese version of the MMSE assessment was administered by trained community doctors and nurses. The MMSE measures the level of cognition in the following aspects: (1) Temporal and spatial orientation: 10 points; (2) Immediate memory: 3 points; (3) Recall: 3 points; (4) Attention: 5 points; (5) Language: 8 points; (6) Visual space: 1 point. The MMSE scores range from 0 to 30, with higher scores indicating higher levels of cognition. In the Chinese population, cognitive impairment was defined as an MMSE score ≤ 17 for those with no education, ≤ 20 for those with 1–6 years of education, or ≤ 24 for those with ≥7 years of education (Guo et al., 1988; Jia et al., 2021).

### 2.4 Closed-eye unipedal standing test

The closed-eye unipedal standing can test a participant's balance strength (Yoshimoto et al., 2016; Büchel et al., 2021). Using a standard stopwatch, participants were allowed to stand naturally or with their arms crossed and eyes closed, standing on one foot with the customary foot and the other leg bent at the knee with the foot off the ground so that the lower leg rested against the knee of the standing leg. The timer was set when the off-ground foot leaves the ground, and the meter was stopped when the off-ground foot hits the ground or the standing foot moves, and the time of standing on one foot with eyes closed was calculated (Rikli and Jones, 2012). Finally, the test was conducted three times, and the average of the three tests was calculated.

### 2.5 Definition of covariates

Covariates consisted of participant's baseline demographic characteristics (age, gender, education, height, weight), health status (hypertension, hyperlipidemia, diabetes, cerebrovascular accident, coronary artery disease, eye and ear disease), and health-related behaviors (smoking status and physical exercise) at baseline. Hypertension, hyperlipidemia, diabetes, cerebrovascular accidents, and coronary artery disease were determined by the question (Do you have a chronic disease diagnosed by a doctor?). Eye and ear diseases were determined by self-reported data and were dichotomous variables. Lack of physical exercise was defined as engaging in moderate-intensity exercise less than three times per week, with each session lasting < 30 min. Smoking was defined as having smoked within the past month, and falls were defined as having fallen within the last 1 year.

### 2.6 Statistical analysis

All analyses were conducted using R (Version 4.2.1) and SPSS (version 26.0) software. Continuous variables were described as mean ± standard deviations (SDs). Categorical variables were expressed by counts and percentages. The elderly were divided into the following three groups according to the tertiles (33.33 and 66.67% digits) of the duration of closed-eye unipedal standing: < 2 s, low group (lowest tertile); 2–4 s, middle group (middle tertile); and ≥4 s, high group (highest tertile). One-way ANOVA and chi-squared tests were used for comparing the differences among these three groups.

Logistic regression models were used to calculate odds ratios (ORs) and 95% confidence intervals (CIs) to evaluate the relationship between closed-eye unipedal standing tertiles and cognitive impairment risks (Zhang and Yu, 1998). To rule out the influence of confounding factors, three models were used: unadjusted; adjusted model 1 (adjusted for age, gender, and years of education), and adjusted model 2 (adjusted for age, gender, years of education, lack of exercise, smoking, and diseases such as hypertension, hyperlipidemia, diabetes, cerebrovascular accident, and coronary artery disease). Meanwhile, we used the restricted cubic spline (RCS) method to flexibly model the potential non-linear relationship between the continuous closed-eye unipedal standing duration and the cognitive impairment risk. We set three knots for the RCS analysis considering the sample size, curve smoothing, and reduced precision due to overfitting. The stratified analysis by baseline characteristics (age, gender, education level, whether lack of exercise or not, and whether falls within a year) were also performed. In this study, multicollinearity statistics (tolerance and variance inflation factors) were computed for each logistic regression model and *P* < 0.05 was considered statistically significant.

## 3 Results

### 3.1 Basic characteristics of participants

Among the 1,652 participants without cognitive impairment at baseline, the mean age was 72.09 ± 4.81 years, and the mean MMSE score was 26.89 ± 2.78. As presented in Table 1, the average of MMSE scores, age, years of education, height and waist circumference, as well as the distribution of gender, hypertension, hyperlipidemia, diabetes, coronary artery disease, eye and ear disease, and whether they lacked exercise, were significantly different among the low, middle, and high closed-eye unipedal standing groups (*P* < 0.05).

**Table 1:** Baseline based on closed-eye unipedal standing participant characteristics.

**Characteristics**	**All (*n* = 1,652)**	**Closed-eye unipedal standing**			***P***
		**Low group (*****n*** = **652)**	**Middle group (*****n*** = **528)**	**High group (*****n*** = **472)**	
MMSE Score in 2015	26.89 ± 2.78	26.03 ± 3.10	27.30 ± 2.46	27.64 ± 2.30	**< 0.001**
Age (years)	72.09 ± 4.81	73.25 ± 5.48	71.38 ± 4.12	71.30 ± 4.17	**< 0.001**
Male (*n*, %)	902 (54.60)	331 (50.77)	298 (56.44)	273 (57.84)	**0.037**
Education (years)	3.61 ± 2.80	3.27 ± 2.84	3.70 ± 2.73	3.97 ± 2.79	**< 0.001**
Height (cm)	161.19 ± 8.01	160.23 ± 7.97	161.90 ± 7.95	161.73 ± 8.02	**< 0.001**
Weight (kg)	63.95 ± 10.72	63.50 ± 10.73	64.07 ± 10.63	64.43 ± 10.81	0.336
BMI (kg/m^2^)	24.58 ± 3.59	24.72 ± 3.75	24.40 ± 3.40	24.60 ± 3.59	0.299
Waist circumference (cm)	87.91 ± 9.84	89.14 ± 10.27	87.66 ± 9.02	86.49 ± 9.93	**< 0.001**
Hip circumference (cm)	96.82 ± 8.49	97.10 ± 8.73	97.03 ± 7.77	96.19 ± 8.90	0.162
Diabetes, yes (*n*, %)	746 (45.16)	266 (40.80)	248 (46.97)	232 (49.15)	**0.013**
Hypertension, yes (*n*, %)	260 (15.74)	75 (11.50)	92 (17.42)	93 (19.70)	**< 0.001**
Hyperlipidemia, yes (*n*, %)	213 (12.89)	104 (15.95)	61 (11.55)	48 (10.17)	**0.009**
Cerebrovascular accident, yes (*n*, %)	233 (14.10)	100 (15.34)	68 (12.88)	65 (13.77)	0.469
Coronary artery disease, yes (*n*, %)	71 (4.30)	38 (5.83)	22 (4.17)	11 (2.33)	**0.017**
Eye and ear diseases, yes (*n*, %)	526 (31.84)	231 (35.43)	164 (31.06)	131 (27.75)	**0.022**
Smoking, no (*n*, %)	1,331 (80.57)	537 (82.36)	410 (77.65)	384 (81.36)	0.111
Falls within 1 year, yes (*n*, %)	164 (9.93)	75 (11.50)	54 (10.23)	35 (7.41)	0.149
Lack of exercise, yes (*n*, %)	353 (21.37)	181 (27.76)	106 (20.08)	66 (13.98)	**< 0.001**

The bold values indicated *P* < 0.05.

### 3.2 Basic characteristics of participants between the cognitive impairment group and the normal group

After 7 years of follow-up, 880 elderlies were included. Among them, 155 (17.61%) participants satisfied the diagnostic criteria for cognitive impairment. As presented in Table 2, the cognitive impairment group was older, had higher education level, and lower height and weight than the normal cognitive group (*P* < 0.05). Significant differences were found between the two groups in terms of lack of exercise (30.32 vs. 20.69%, *P* < 0.001). The elderly in the low closed-eye unipedal standing tertile had a higher prevalence of cognitive impairment compared to the middle and high groups (*P* < 0.01).

**Table 2:** Participant characteristics based on cognitive impairment at follow-up.

**Characteristics**	**All (*n* = 880)**	**Cognitive impairment (*n* = 155)**	**Normal cognition (*n* = 725)**	***P***
Age (years)	78.34 ± 4.21	79.26 ± 4.67	78.14 ± 4.09	**< 0.001**
Male (*n*, %)	465 (52.84)	73 (47.10)	392 (54.07)	0.114
Education (years)	3.76 ± 2.79	4.59 ± 3.20	3.60 ± 2.67	**< 0.001**
Height (cm)	161.25 ± 7.93	159.05 ± 8.68	161.73 ± 7.68	**< 0.001**
Weight (kg)	64.13 ± 10.61	62.49 ± 9.97	64.48 ± 10.73	**0.027**
BMI (kg/m^2^)	24.64 ± 3.66	24.73 ± 3.78	24.62 ± 3.63	0.754
Waist circumference (cm)	87.71 ± 9.73	87.47 ± 9.72	87.77 ± 9.74	0.730
Hip circumference (cm)	96.66 ± 8.35	96.59 ± 8.26	96.68 ± 8.37	0.908
Diabetes, yes (*n*, %)	394 (44.77)	75 (48.39)	319 (44.00)	0.319
Hypertension, yes (*n*, %)	145 (16.48)	25 (16.13)	120 (16.55)	0.898
Hyperlipidemia, yes (*n*, %)	109 (12.39)	24 (15.48)	85 (11.72)	0.197
Cerebrovascular accident, yes (*n*, %)	109 (12.39)	15 (9.68)	94 (12.97)	0.259
Coronary artery disease, yes (*n*, %)	29 (3.30)	4 (2.58)	25 (3.45)	0.583
Eye and ear diseases, yes (*n*, %)	269 (30.57)	44 (28.39)	225 (31.03)	0.516
Smoking, no (*n*, %)	715 (81.25)	127 (81.93)	588 (76.97)	0.810
Falls within 1 year, yes (*n*, %)	83 (9.43)	10 (6.45)	73 (10.07)	0.225
Lack of exercise, yes (*n*, %)	197 (22.39)	47 (30.32)	150 (20.69)	**< 0.001**
**Closed-eye unipedal standing**				
Low group (*n*, %)	344 (39.09)	79 (50.97)	265 (36.55)	**0.003**
Middle group (*n*, %)	270 (30.68)	41 (26.45)	229 (31.59)	
High group (*n*, %)	266 (30.23)	35 (22.58)	231 (31.86)	

The bold values indicated *P* < 0.05.

### 3.3 The relationship between closed-eye unipedal standing and cognitive impairment

The scatterplot between participants' closed-eye unipedal standing time and MMSE scores showed a non-linear relationship, as shown in Supplementary Figure 1. Considering the low group as the reference group, the cognitive impairment risks in the middle (OR = 0.601, 95% CI: 0.396–0.911) and high (OR = 0.508, 95% CI: 0.329–0.785) groups were significantly lower. After adjusting for covariates, results were similar to those before adjustment (Table 3).

**Table 3:** Association between closed-eye unipedal standing and cognitive impairment.

	**B**	**S. E**	**Wald**	***P***	**OR**	**95% CI for OR**	
						**Lower**	**Upper**
**Unadjusted**							
Low group	Ref				Ref		
Middle group	−0.510	0.213	5.753	**0.016**	0.601	0.396	0.911
High group	−0.677	0.222	9.285	**0.002**	0.508	0.329	0.785
**Model 1**							
Low group	Ref				Ref		
Middle group	−0.493	0.227	4.716	**0.030**	0.611	0.391	0.953
High group	−0.639	0.234	7.475	**0.006**	0.528	0.334	0.834
**Model 2**							
Low group	Ref				Ref		
Middle group	−0.452	0.231	3.828	**0.050**	0.636	0.404	1.000
High group	−0.600	0.240	6.255	**0.019**	0.568	0.354	0.911

**Model 1:** adjusted for age, gender, and education.

**Model 2:** adjusted for age, gender, education, height, weight, diabetes, hypertension, hyperlipidemia, cerebrovascular accident, coronary artery disease, smoking, falls within 1 year, and lack of exercise.

The bold values indicated *P* < 0.05.

**Figure 2:**
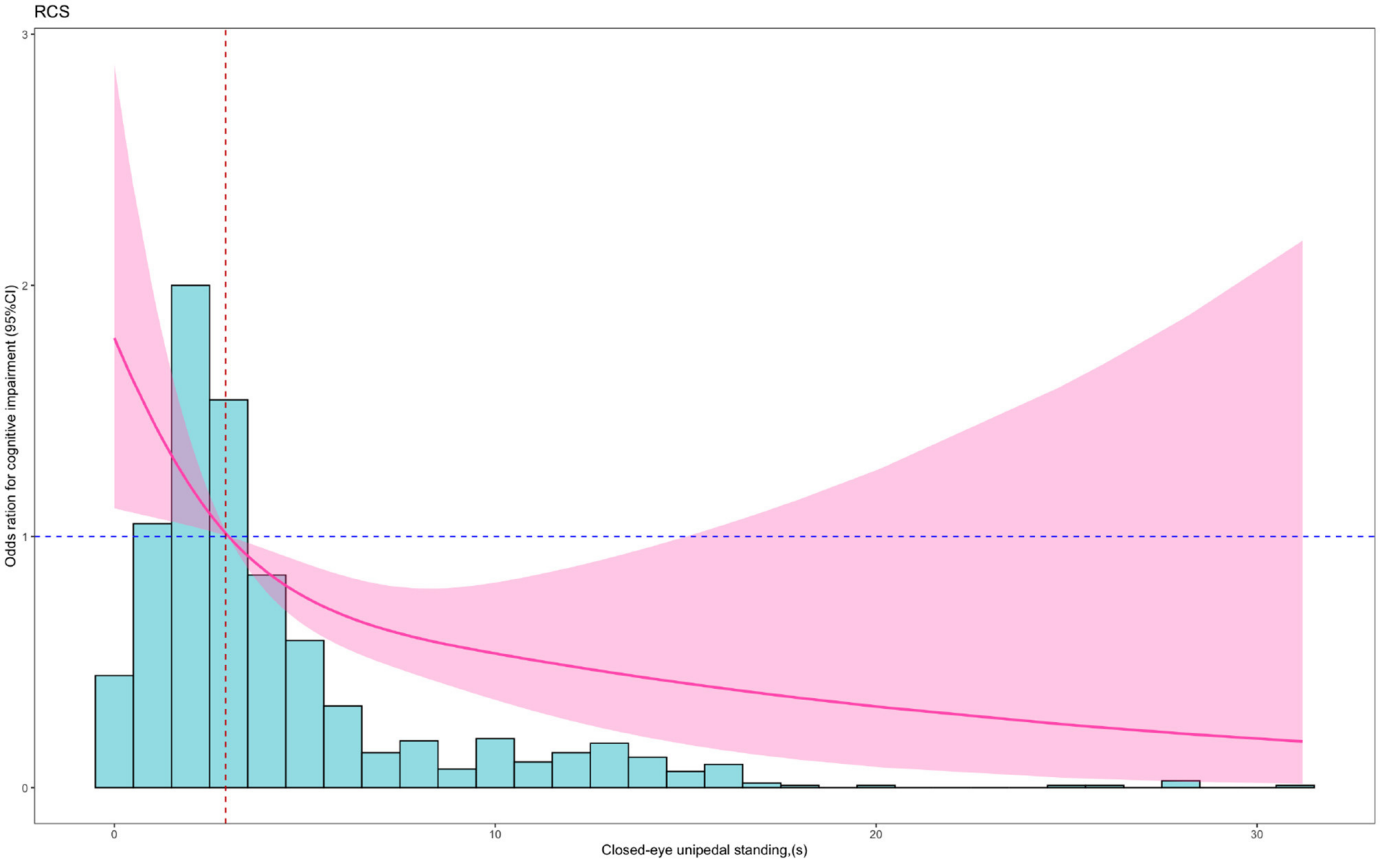
RCS plot of the association between closed-eye unipedal standing and cognitive impairment. Red solid lines showed the odds ratios, and red shading showed the 95% confidence interval. The analysis was adjusted for age, sex, education, height, weight, diabetes, hypertension, hyperlipidemia, cerebral stroke, coronary artery disease, and lack of exercise.

To flexibly model and visualize the relationship between continuous closed-eye unipedal standing and the risk of cognitive impairment, we further performed the regression analysis using RCS. The results were consistent with the overall results. Within a short period of closed-eye unipedal standing of ~2.920 s, there was no protective effect on reducing the risk of cognitive impairment in older adults, and the risk of cognitive impairment gradually decreased as the duration of closed-eye unipedal standing increased (*P*_non − linear_ = 0.177) (Figure 2).

### 3.4 Stratified analysis of the association between closed-eye unipedal standing and cognitive impairment

**Figure 3:**
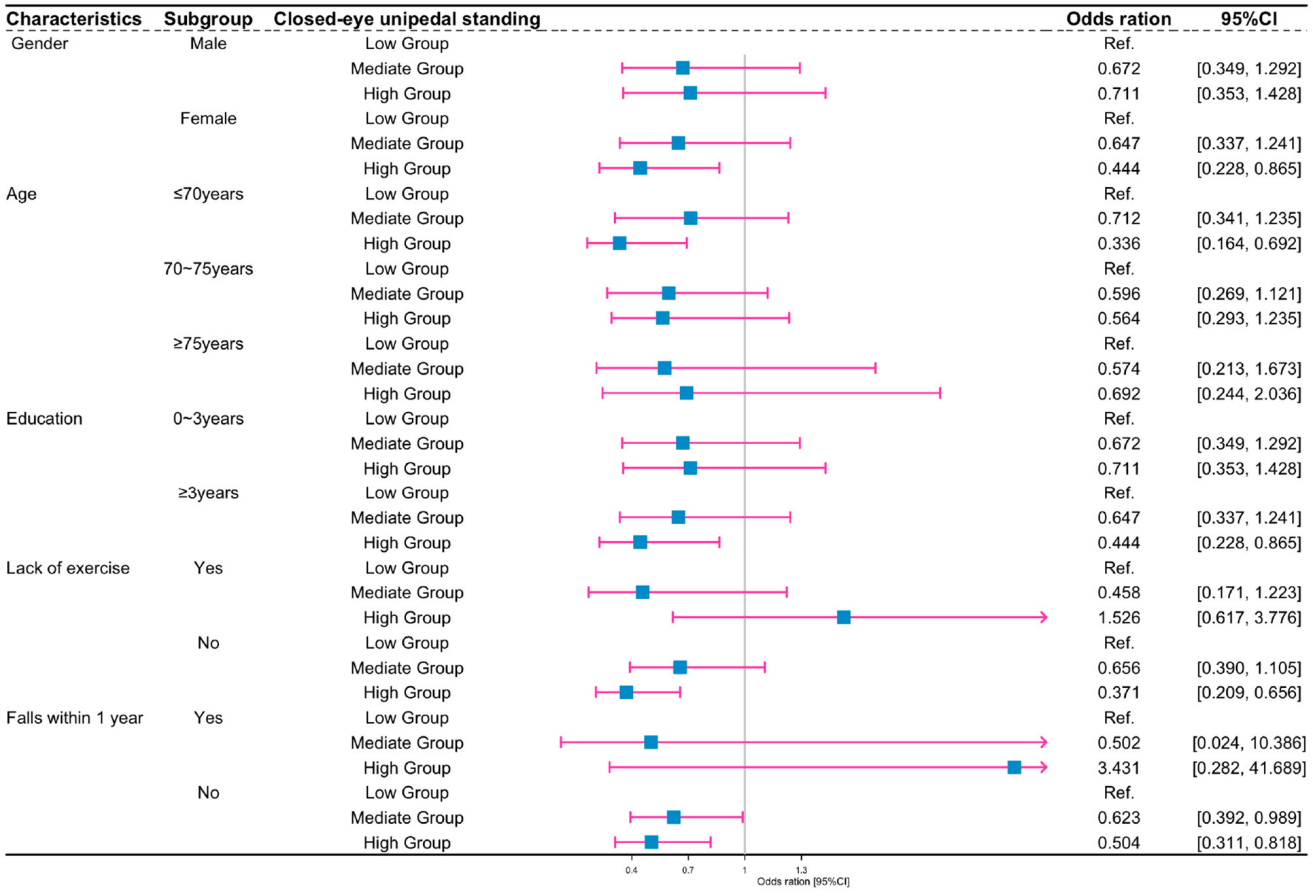
Subgroup analysis of the association between closed-eye unipedal standing and cognitive impairment according to participant characteristics. **Gender group:** adjusted for age, education, height, weight, diabetes, hypertension, hyperlipidemia, cerebrovascular accident, coronary artery disease, smoking, falls within 1 year, and lack of exercise. **Age group:** adjusted for gender, education, height, weight, diabetes, hypertension, hyperlipidemia, cerebrovascular accident, coronary artery disease, smoking, falls within 1 year, and lack of exercise. **Education group:** adjusted for age, gender, height, weight, diabetes, hypertension, hyperlipidemia, cerebrovascular accident, coronary artery disease, smoking, falls within 1 year, and lack of exercise. **Lack-of-exercise group:** adjusted for age, gender, education, height, weight, diabetes, hypertension, hyperlipidemia, cerebrovascular accident, coronary artery disease, smoking, and falls within 1 year. **Falls-within-1-year group:** adjusted for age, gender, education, height, weight, diabetes, hypertension, hyperlipidemia, cerebrovascular accident, coronary artery disease, smoking, and lack of exercise.

Figure 3 illustrates the stratified analysis of the association between closed-eye unipedal standing groups and cognitive impairment risk by gender, age, years of education, whether lack of exercise or not and whether falls within 1 year. The results showed that for women, ≤ 70 years old, with ≥ 3 years of education, without lack of exercise and without falls within 1 year subgroup, the participants with the high group of closed-eye unipedal standing exhibited a significant decrease in the cognitive impairment risk. However, there were no statistically significant associations between the middle group of closed-eye unipedal standing and cognitive impairment risk in any of the different subgroups.

## 4 Discussion

To the best of our knowledge, the relationship between closed-eye unipedal standing and the risk of cognitive impairment was explored for the first time in the Chinese elderly population. By following up on participants for 7 years, we found that the cognitive impairment risks in middle and high closed-eye unipedal standing groups were significantly lower. Furthermore, the results of the RCS analysis indicated that, when the duration exceeded ~2.920 s, the risk of cognitive impairment in the elderly decreased with increasing duration of closed-eye unipedal standing. These relationships remained significant after adjusting for confounders.

After 7 years of follow-up, the follow-up rate of the elderly was only 53.27%, which might limit the extrapolation and accuracy of our results. The reasons for missing follow-up included elderly individuals relocating with their offspring, death, or unwillingness to visit the hospital or accept the doctor's visit due to coronavirus disease (COVID-19), and so on. Finally, the incidence rate of cognitive impairment in the elderly was 17.61%. In the Chinese Longitudinal Healthy Longevity Survey (CLHLS), Zhang et al. (2022) found that, after 3 years of follow-up, 14.4% were diagnosed with cognitive impairment, which is lower than that observed in this study, possibly because the diagnostic criteria for cognitive impairment (MMSE scores < 24) were different and the follow-up interval years were shorter.

During closed-eye unipedal standing, cooperative work of multiple physiological systems is essential. Among these, the neurological system relies on intrinsic sensations, such as proprioceptors and the vestibular system, to visualize the position and movement of the body. Simultaneously, the muscle system and articulations perform an essential role in maintaining the body's stability by adjusting muscle tensions (Serra-Añó et al., 2015; Afschrift et al., 2021). Existing evidence suggests that controlling body posture can improve cognitive performance by stimulating the corresponding functional areas of the brain (Henry and Baudry, 2019). It can be speculated that closed-eye unipedal standing may be much difficult for people with cognitive impairment. This is because they might struggle to effectively perceive their body's position and posture, thereby facing difficulties in making timely adjustments to maintain balance.

Our results showed that, when compared with the low tertile group, the risk of cognitive impairment was 0.601 and 0.508 times higher in the middle and high closed-eye unipedal standing tertile groups, respectively. The RCS results also indicated that higher closed-eye unipedal standing duration was protective against the risk of cognitive impairment in older adults. In spite of reaching a certain duration, the CI included 1, which might be due to a potentially insufficient sample size combined with other confounding factors, such as dietary habits. Similarly, many researchers, such as Rolland et al. (2009) and Tabara et al. (2015), have shown that the single-leg balance standing test can predict cognitive decline in the elderly, possibly due to parietal and hippocampal dysfunction which can lead to memory loss and visual orientation deficits. Sugihara, on the other hand, revealed that activation of the left and right prefrontal cortex was observed during one-legged standing in the absence of a cognitive function task; however, this activation was altered in aging, cognitive impairment, and Parkinson's syndrome subjects (Sugihara et al., 2021). Hence, the closed-eye unipedal standing duration might be a predictive index for cognitive impairment in the elderly.

Furthermore, the histogram in our study showed that the majority of the elderly performed the closed-eye unipedal standing at a lower level. Previous studies have concluded that intensive practice of closed-eye unipedal standing may help activate and improve areas of the brain associated with balance and spatial cognition, thereby facilitating connections and information transfer between neurons (Bustillo-Casero et al., 2017; Papegaaij et al., 2017). Involving the elderly in exercise to improve balance and lower limb muscle strength to prolong closed-eye unipedal standing may be helpful to delay the development of cognitive impairment. Hence, the closed-eye unipedal standing, being a simple and adaptable method, can serve as a predictive index for cognitive impairment in the elderly. Additionally, it might also be considered an intervention method to prevent cognitive dysfunction. Further intervention studies are necessary to confirm this hypothesis.

The stratified analysis showed that, after adjusting for confounders, the risk of cognitive impairment in the female elderly was much lower than the male elderly. It is possible that the female elderly individuals are more prone to mood swings, insomnia, irritability and anxiety, which may affect the brain functions to a certain extent (Livingston et al., 2020). In addition, consistent with the previous findings by Ahn and Kim (2023) and Nagata et al. (2023), closed-eye unipedal standing reduces the risk of cognitive impairment in the elderly in the exercise group but not in the lack of exercise group. With increasing age, certain elderly people lose their ability to balance, along with degenerative changes in the brain tissue, such as amyloidosis and demyelination in nerve cells (Lourenco et al., 2018; Omura et al., 2020; Yang et al., 2023). Furthermore, decreased levels of the acetylcholine mediator contribute to reduced brain function, which in turn leads to the possibility that closed-eye unipedal standing may have a lesser impact on the risk of cognitive impairment in people over 75 years of age.

## 5 Limitations

While this study has important theoretical and practical implications, it also has certain limitations and points to areas for further exploration in the future. First, the rate of loss to follow-up was relatively high. The follow-up assessment was conducted only once. The evolution of the mean cognitive impairment scores over time could not be depicted. Second, the recruited participants were homogeneously aged and sourced from diverse geographical regions within Wuhan City. The definition of cognitive impairment depended only on MMSE scores. Due to sample size limitations, the influence of baseline MMSE scores on the effect of closed-eye unipedal standing duration on the risk of cognitive impairment was not completely excluded. These limitations potentially limited the generalizability of the findings to the broader elderly Chinese population. Finally, the study did not account for all the potential confounding factors, such as diet, sleep, and so on. In the future, we will continue to conduct further rigorous prospective and intervention studies to reveal the relationship and mechanisms between closed-eye unipedal standing and cognitive impairment.

## 6 Conclusion

Our results showed that, closed-eye unipedal standing in the elderly was linearly and negatively associated with cognitive impairment. The risk of developing cognitive impairment was decreased when the duration of closed-eye unipedal standing exceeded approximately 2.920 s. The closed-eye unipedal standing duration might be a predictive index for cognitive impairment in the elderly. Further intervention studies are necessary to confirm the protective effects and mechanisms of closed-eye unipedal standing on cognitive impairment in the elderly.

## Data availability statement

The raw data supporting the conclusions of this article will be made available by the authors, without undue reservation.

## Ethics statement

The studies involving humans were approved by the Institutional Review Board of Wuhan University of Science Technology (Number: 2023120). The studies were conducted in accordance with the local legislation and institutional requirements. The participants provided their written informed consent to participate in this study.

## Author contributions

SW: Conceptualization, Data curation, Formal analysis, Investigation, Software, Writing – original draft, Writing – review & editing. PG: Writing – original draft, Writing – review & editing. CH: Software, Writing – review & editing. YZ: Data curation, Writing – review & editing. BX: Investigation, Writing – review & editing. JZ: Data curation, Writing – review & editing. FZ: Data curation, Writing – review & editing. XX: Conceptualization, Writing – review & editing. YG: Data curation, Writing – original draft. MY: Conceptualization, Investigation, Writing – review & editing.
