# Supplementary material for: The association between closed-eye unipedal standing and the risk of cognitive impairment in the elderly: a 7-year community-based cohort study in Wuhan, China
Source: Front Aging Neurosci. 2024 Jan 26;16:1308151. doi: 10.3389/fnagi.2024.1308151 (PMC10853411; doi:10.3389/fnagi.2024.1308151)
Supplement: Supplementary file 1 [file Data_Sheet_1.docx]

**Supplementary Material**


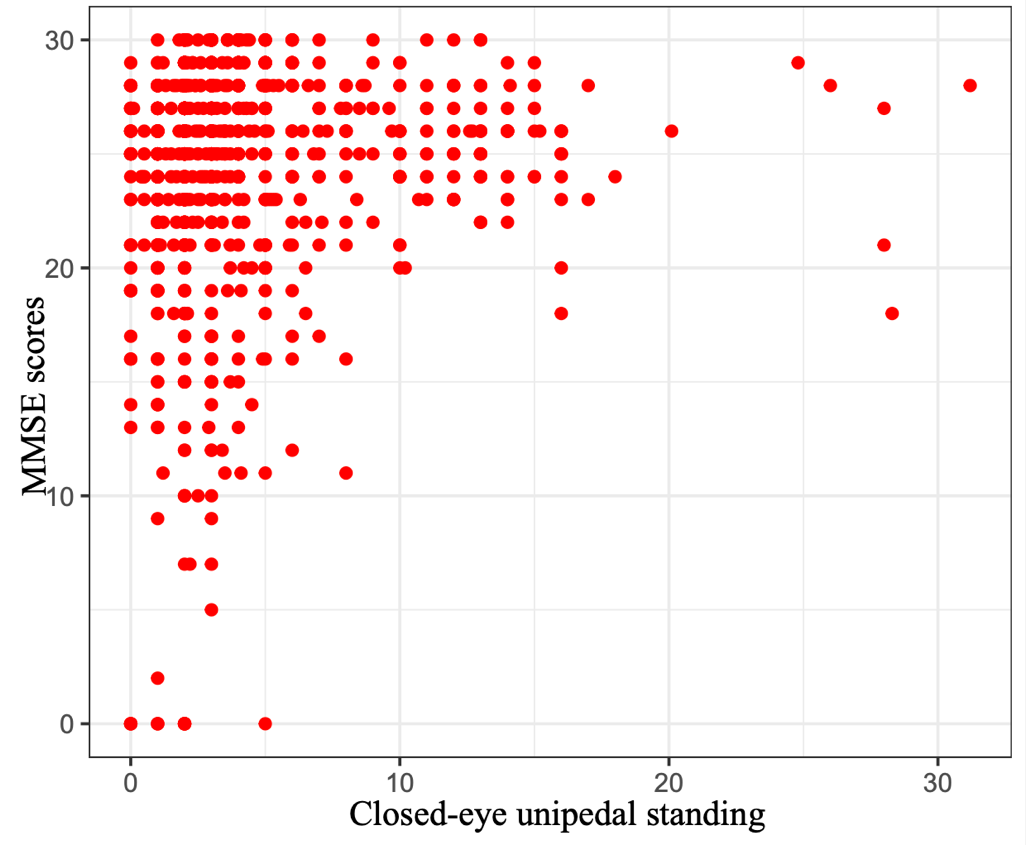


**Supplementary Fig1：Scatterplot of closed-eye unipedal standing and MMSE scores.**
